# Proteomic Analysis Revealed the Potential Role of MAGE-D2 in the Therapeutic Targeting of Triple-Negative Breast Cancer

**DOI:** 10.1016/j.mcpro.2023.100703

**Published:** 2023-12-20

**Authors:** Xiaoyu Shi, Chunyan Liu, Weimin Zheng, Xiao Cao, Wan Li, Dongxue Zhang, Jianhua Zhu, Xian Zhang, Yun Chen

**Affiliations:** 1 School of Pharmacy, Nanjing Medical University, Nanjing, China; 2 State Key Laboratory of Reproductive Medicine and Offspring Health, Nanjing, China; 3 Key Laboratory of Cardiovascular & Cerebrovascular Medicine, Nanjing, China

**Keywords:** MAGE-D2, proteomic analysis, triple-negative breast cancer, cell proliferation and metastasis, potential therapeutic target

## Abstract

Among all the molecular subtypes of breast cancer, triple-negative breast cancer (TNBC) is the most aggressive one. Currently, the clinical prognosis of TNBC is poor because there is still no effective therapeutic target. Here, we carried out a combined proteomic analysis involving bioinformatic analysis of the proteome database, label-free quantitative proteomics, and immunoprecipitation (IP) coupled with mass spectrometry (MS) to explore potential therapeutic targets for TNBC. The results of bioinformatic analysis showed an overexpression of MAGE-D2 (melanoma antigen family D2) in TNBC. *In vivo* and *in vitro* experiments revealed that MAGE-D2 overexpression could promote cell proliferation and metastasis. Furthermore, label-free quantitative proteomics revealed that MAGE-D2 acted as a cancer-promoting factor by activating the PI3K–AKT pathway. Moreover, the outcomes of IP–MS and cross-linking IP–MS demonstrated that MAGE-D2 could interact with Hsp70 and prevent Hsp70 degradation, but evidence for their direct interaction is still lacking. Nevertheless, MAGE-D2 is a potential therapeutic target for TNBC, and blocking MAGE-D2 may have important therapeutic implications.

## Highlights

• Proteomic analysis involves bioinformatics, label-free quantification, IP–MS, XL–MS.
• MAGE-D2 promotes cell proliferation and metastasis by activating PI3K–AKT pathway.
• MAGE-D2 could interact with Hsp70 and inhibit its degradation.
• MAGE-D2 is overexpressed in TNBC and is a potential therapeutic target for TNBC.

## Graphical Abstract

**Figure:**
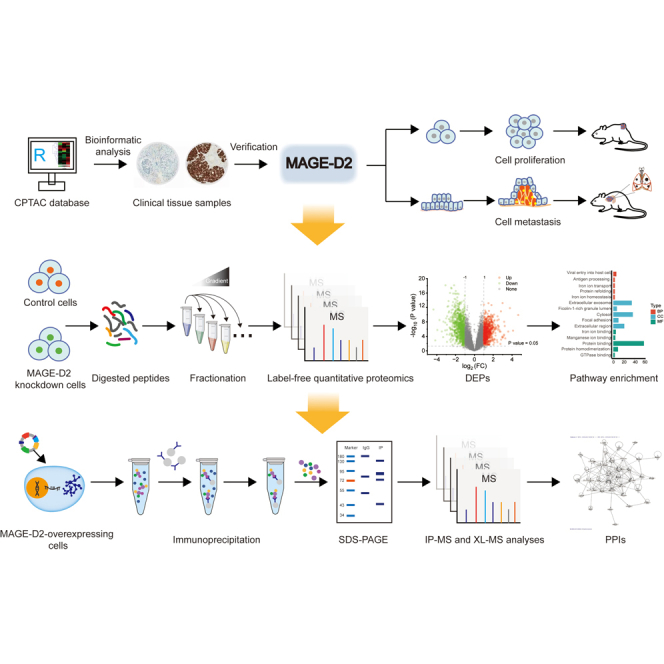

## In Brief

A combined proteomic analysis involving bioinformatic analysis, label-free quantitative proteomics, IP–MS, and XL–MS was carried out to reveal the potential of MAGE-D2 as a therapeutic target for TNBC. The results showed an overexpression of MAGE-D2 in TNBC, which could promote cell proliferation and metastasis by activating the PI3K–AKT pathway. Furthermore, MAGE-D2 could interact with Hsp70 and protect Hsp70 from degradation. Thus, blocking MAGE-D2 may have important therapeutic implications.

Triple-negative breast cancer (TNBC) is a specific subtype of breast cancer in which hormone receptors, such as estrogen receptor, progesterone receptor, and human epidermal growth factor receptor 2, cannot be detected (1). TNBC is distinguished from other breast cancer subtypes by its high invasiveness, poor prognosis, and high recurrence rate (2). Because hormone receptors and growth factor receptors are absent in TNBC, molecular targeted therapies for this disease are limited. Therefore, elucidation of the molecular mechanisms of TNBC and identification of potential therapeutic targets are urgently needed for TNBC.

To date, a series of studies have focused on the molecular mechanisms underlying TNBC and discovered several potential therapeutic targets. For example, there is evidence indicating that the PI3K–AKT pathway is altered in TNBC. Inhibition of this pathway has been explored for the treatment of TNBC (3). In addition, a member of the heat shock protein family, Hsp70, is overexpressed in TNBC, and there is evidence indicating that Hsp70 plays an important role in cancer cell proliferation and metastasis (4, 5). Nevertheless, a comprehensive screening of factors regulating TNBC occurrence and development has not been performed, and the precise mechanism underlying the discovered pathways and molecules is still unclear. Therefore, useful tools should be adopted to uncover more information.

Proteomic analysis plays a critical role in illustrating mechanisms and revealing targets. This type of analysis can be used to explore the deep and comprehensive landscape of diseases. Currently, the most comprehensive and adaptable tool in large-scale proteomics is mass spectrometry (MS). Among various MS-based proteomics strategies, label-free quantitative proteomics has high analytical depth, dynamic range, and quantitative capability (6). It offers a straightforward and objective platform for thorough protein expression profiling of biological samples to identify biomarkers and therapeutic targets (7). In addition, thousands of protein datasets are available in data repositories such as the Clinical Proteomic Tumor Analysis Consortium (8), which facilitates the discovery of target proteins of interest through bioinformatic analysis. To further understand the molecular mechanism of target proteins and detect their interacting proteins, another two proteomics strategies including immunoprecipitation–MS (IP–MS) and chemical cross-linking coupled with MS (XL–MS) have been employed to effectively identify protein‒protein interactions (PPIs) (9). This information can be used to clarify the functions and roles of target proteins in disease.

In this study, integrated proteomic analysis was performed to reveal potential therapeutic target proteins in TNBC. A member of the melanoma antigen (MAGE) family, MAGE-D2, was identified as a candidate target through bioinformatic analysis and histological validation. Then, the shRNA (siRNA) or overexpression plasmid of MAGE-D2 was transfected into TNBC cells to knockdown or overexpress the target protein, respectively. The function of MAGE-D2 was evaluated by both *in vitro* and *in vivo* experiments. Moreover, label-free quantitative proteomics was applied to confirm the expression of MAGE-D2 in TNBC. Furthermore, we conducted IP–MS and XL–MS to explore the interacting proteins of MAGE-D2. Finally, rescue experiments were carried out to investigate the effect of the PPI on the growth and metastasis of TNBC.

## Experimental Procedures

### Clinical Sample Collection

A total of 50 paired TNBC cancer samples were obtained from Jiangsu Cancer Hospital and approval of the Institutional Review Board (2019[022]). The corresponding patients were enrolled between January 2019 and August 2021, meeting the inclusion criteria as follows: (1) 18 years and older; (2) pathologically diagnosed with TNBC without preoperative chemotherapy or radiotherapy; and (3) the absence of other concomitant or previous malignant disease within 5 years. Informed consent was obtained from each patient. We did not list the information that identifies individuals in this study.

### Cell Culture

Four TNBC cell lines (MDA-MB-231, MDA-MB-157, MDA-MB-468, and HCC1937 abbreviated 231, 157, 468, and 1937, respectively) were purchased from the Cell Resource Center of the Chinese Academy of Medical Sciences. Normal breast cell line MCF-10A was kindly donated by Dr Ziyi Fu at Nanjing Medical University. These cell lines were authenticated by short tandem repeat analysis before proteomic experiments were conducted. The 231 cells and 157 cells were cultured in RPMI1640 medium supplemented with 10% fetal bovine serum at 37 °C under a 5% CO_2_ atmosphere, and 468 and 1937 cells were cultured in Dulbecco's modified Eagle's medium supplemented with 10% fetal bovine serum at 37 °C under a 5% CO_2_ atmosphere. These cells were regarded as control cells. MCF-10A normal cells were routinely maintained in MEGM kit (Lonza/Clonetics; CC-3150) supplemented with 100 ng/ml cholera toxin at 37 °C in 5% CO_2_.

### MS-Based Label-Free Quantitative Proteomics

MAGE-D2 knockdown cells and control cells were collected for analysis. For denaturation, proteins were soaked in 8.00 M urea and heated to 25 °C for 1 h. At 20 °C, proteins were precipitated with acetone for 2 h. After 10.0 mM DTT was added at 55 °C for 90 min, 50.0 mM indole-3-acetic acid was added at room temperature for 30 min in the dark. Trypsin was subsequently added to the protein solution at a 1:20 ratio for 12 h. Then, peptides were desalted and separated into 30 fractions using a C18 column with a 0.2 ml/min rate and a gradient of 55 min. Then, the 30 fractions were pooled into 5 fractions. After being diluted in 0.1% formic acid, the peptides were immediately put into a reversed-phase analytical column for EASY-nLC 1200 HPLC coupled with Orbitrap Fusion Lumos analysis.

### XL–MS Analysis

After transfection, MAGE-D2-overexpressing 231 cells were collected, and total protein was extracted according to the steps in label-free quantitative proteomics. The total protein was incubated with MAGE-D2 antibody overnight, and then the complex was mixed with agarose beads and further concentrated to 1.00 mM. Disuccinimidyl suberate (1.50 mM) was used to crosslink the supernatant proteins, and the cross-linking process was slowed using 50.0 mM Tris–HCl (pH = 7.5). The proteins were resuspended in NH_4_HCO_3_ (pH = 8.5) and incubated with Lys-C for 4 h and trypsin (1:20) for 12 h. EASY-nLC™ 1200 HPLC coupled with Orbitrap Fusion Lumos was used for liquid chromatography–tandem MS (LC-MS/MS) analysis. The cross-linked peptides were located by using the program pLink2.0 with precursor and fragment ion mass precision at 20 ppm. After applying a 5% false discovery rate (FDR) cutoff for the results at the spectrum level, the results were filtered.

### LC‒MS/MS Analysis

Peptides were dissolved and injected into a UPLC system. The gradient was from 3% to 5% over 5 s, 5% to 15% over 23 min and 55 s, 15% to 28% over 21 min, 28% to 38% in 7.5 min, a climb to 100% in 5 s, and a holding period at 100% for the final 12 min and 25 s. The peptides were detected by MS in an Orbitrap Fusion Lumos connected with UPLC from *m/z* 350 to 1500 at a resolution of 60,000, automatic gain control target of 2 × 10^5^, and 20 ms injection time. To disassemble the peptides, higher-energy collision dissociation was performed, and the Orbitrap cell was used to identify the fragments. The next 90 s of sequencing did not include any of the chosen precursor ions.

### Database Searching and Data Filtering

MS raw files were processed using MaxQuant, version 1.6.3.3 (Max Planck Institute of Biochemistry), and 203,835 UniProtKB human protein entries published on February 11, 2022 were used for database searching (Supplemental Table S1). The search was conducted using the following criteria: trypsin was used to fully digest the samples; up to three missed cleavages; 20 ppm precursor mass tolerance; 0.025 Da product ion mass tolerance; carbamidomethylation of cysteine as a fixed modification, and methionine oxidation as a variable modification. For peptide and protein identification, the global FDR cutoff was set at 0.01. Perseus (version 1.5.3.2, Max Planck Institute of Biochemistry) was used to view and further analyze the data. Protein abundance was calculated using label-free quantitation intensity.

### Experimental Design and Statistical Rationale

Three biological replicates were carried out for label-free quantitative proteomic analysis. Proteins identified in all three replicates with “peptides” ≥3 and “unique peptides” >1, and 1% FDR at peptide and protein levels for each cell line were considered. In MAGE-D2 IP–MS experiments, proteins identified in at least two replicates with “peptides” ≥3 and “unique peptides” >3, and 1% FDR at peptide and protein levels were considered. In MAGE-D2 XL–MS experiments, proteins identified in all three replicates with “peptides” ≥ 3 and “unique peptides” >3 and 1% FDR at peptide and protein levels were considered.

Statistical analysis was performed using SPSS 17.0 software (International Business Machines Corporation). Data are presented as means ± SDs. A *p* value <0.05 was considered to indicate statistical significance. For all histology and immunofluorescence quantification, the researchers were blinded. Student's *t* test was used to compare two groups. For comparison of various groups, ANOVA was used. Survival analysis by Kaplan–Meier curves and log-rank (Mantel–Cox) test was performed. After protein classification by Gene Ontology (GO) annotation, two-tailed Fisher's exact test was applied to each category to evaluate whether the differentially expressed protein (DEP) was enriched against all identified proteins. GO terms with a corrected *p* value < 0.05 were considered significantly enriched.

### Ethical Approval

All procedures performed in studies involving human participants were in accordance with the ethical standards of the institutional and/or national research committee and with the 1964 Helsinki declaration and its later amendments or comparable ethical standards.

## Results

### Bioinformatic Analysis of the Proteome Database Reveals that MAGE-D2 is Highly Expressed in TNBC

**Fig. 1:**
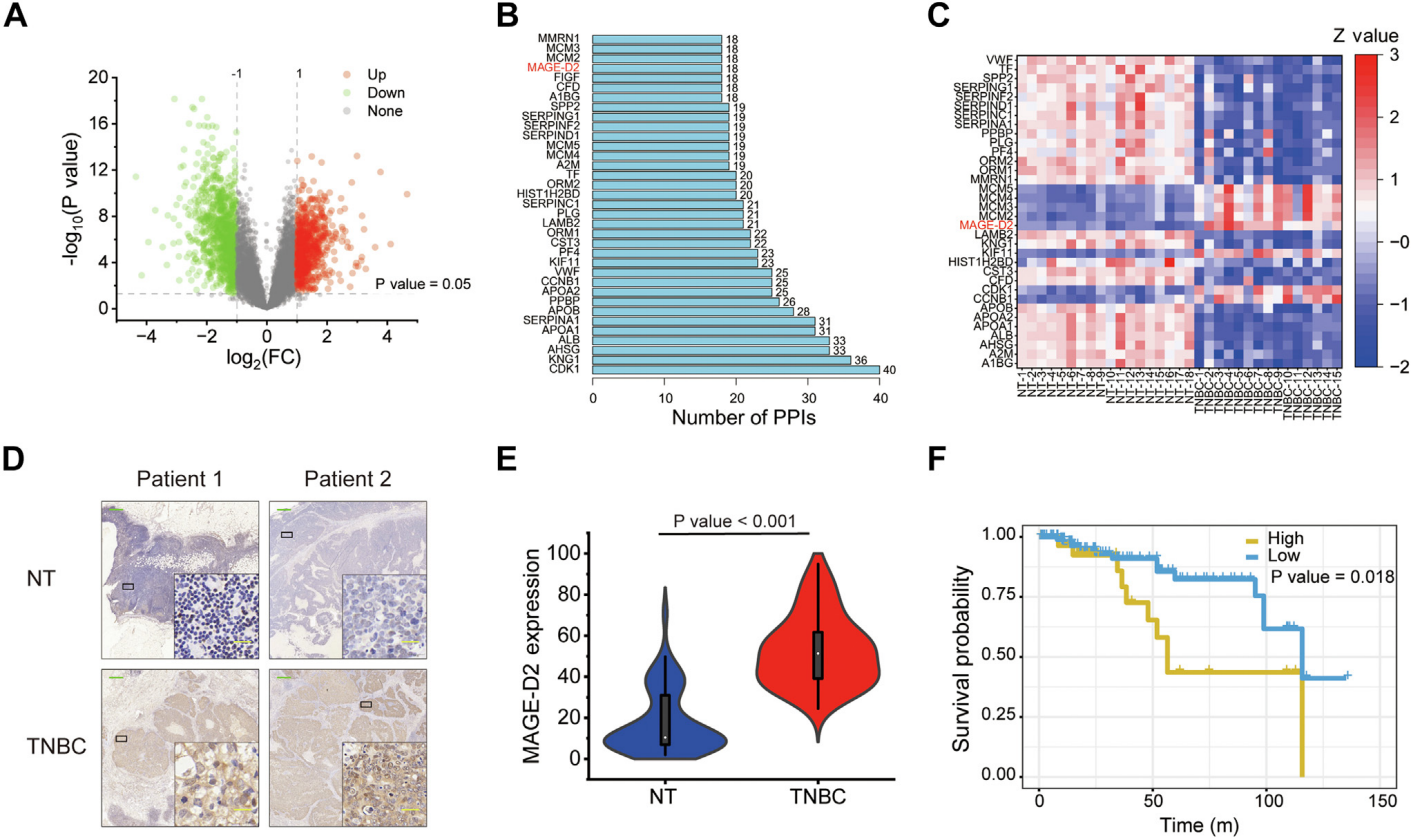
**Proteomic and bioinformatic analyses revealed MAGE-D2 overexpression in TNBC.***A*, volcano plot of all DEPs from CPTAC database. It shows upregulated proteins, downregulated proteins, and unchanged proteins as *red dots*, *blue dots*, and *gray dots*, respectively. *B*, bar chart of top 35 hub proteins and their corresponding node degrees analyzed by STRING database. Eight overlapping proteins are highlighted in *red*. *C*, heatmap of the expression of top 35 node proteins in TNBC patients from CPTAC database. *D*, IHC analysis of MAGE-D2 in TNBC cancer tissue and adjacent tissue (NT) as control. Scale bar represents 500 μm (*green*) and 20 μm (*yellow*). *E*, the expression of MAGE-D2 in TNBC cancer tissue and NT *via* IHC (n = 50). *F*, Kaplan‒Meier survival curves of the TNBC patients stratified by mRNA expression of MAGE-D2 (n = 879). Statistical significance was calculated using a two-tailed unpaired Student's *t* test in *E* and log-rank (Mantel–Cox) test in *F*. CPTAC, Clinical Proteomic Tumor Analysis Consortium; DEP, differentially expressed protein; IHC, immunohistochemical; MAGE-D2, melanoma antigen family D2; TNBC, triple-negative breast cancer.

First, we analyzed the proteomic data of TNBC tissue samples from 15 participants and adjacent breast tissue samples from 18 participants prospectively collected by the Clinical Proteomic Tumor Analysis Consortium. After data processing and correction, 587 DEPs (*p* < 0.05, log_2_ fold change >1 or <−1), including 212 upregulated DEPs and 375 downregulated DEPs, were identified (Fig. 1*A*). Then, the software STRING designated by Global Biodata Coalition and ELIXIR. was used to analyze the interaction between the 587 DEPs with the *Homo sapiens* background and a standard confidence score of 0.9. After that, we counted and listed the top 35 hub proteins (Fig. 1*B*) and then compared this set of 35 hub proteins with the upregulated DEPs in this study. Finally, we obtained overlapping eight proteins, and these proteins were sorted according to the log_2_ fold change in Table 1. Among them, several proteins have been investigated previously. For example, the inhibitor of KIF11 combined with chemotherapy can lead to a better response in TNBC (10). Cyclin-dependent kinase-1 inhibition can enhance the effect of other treatments on TNBC cells (11). More importantly, MAGE-D2 (also called breast cancer–associated gene 1 protein [BCG-1]) was significantly overexpressed in TNBC (Fig. 1*C*). However, there have been few reports on MAGE-D2 in TNBC or even in cancer thus far. Hence, we focused on MAGE-D2 in this research.

**Table 1:** Comparison of the expression of eight identified proteins in TNBC cancer tissue *versus* adjacent tissue

Spot no.	Entry name	Protein name and synonyms	SwissProt accession no.	Reported functions	log_2_FC	*p*
1	KIF11	Kinesin-like protein	P52732	Motor protein required for establishing a bipolar spindle during mitosis	2.30	3.28 × 10^−7^
2	CDK1	Cyclin-dependent kinase 1	P06493	Promotes G2-M transition and regulates G1 progress and G1-S transition	2.28	2.40 × 10^−7^
3	MAGE-D2	Melanoma-associated antigen D2	Q9UNF1	Tumor antigen	2.05	2.64 × 10^−7^
4	MCM4	DNA replication licensing factor	P33991	Acts as component of the MCM2-7 complex	1.86	4.30 × 10^−7^
5	CCNB1	G2/mitotic specific cyclin-B1	P14635	Essential for the control of the cell cycle at the G2/M transition	1.71	1.36 × 10^−5^
6	MCM2	DNA replication licensing factor	P49736	Acts as component of the MCM2-7 complex	1.70	7.87 × 10^−7^
7	MCM3	DNA replication licensing factor	P25205	Acts as component of the MCM2-7 complex	1.68	1.61 × 10^−6^
8	MCM5	DNA replication licensing factor	P33992	Acts as component of the MCM2-7 complex	1.50	7.32 × 10^−6^

We then carried out immunohistochemical analysis of cancer and adjacent tissue samples of 50 TNBC patients involved in this study to validate this proteomic finding at a clinicopathological level. The results confirmed that MAGE-D2 was highly expressed in cancer tissue compared with adjacent tissue (Fig. 1, *D* and *E*). Furthermore, Kaplan‒Meier survival analysis showed that patients with low MAGE-D2 expression had higher overall survival rates than patients with high MAGE-D2 expression (Fig. 1*F*).

### MAGE-D2 Promotes TNBC Proliferation and Metastasis *In vitro*

Next, we compared the protein expression of MAGE-D2 in four TNBC cell lines (231, 157, 468, and 1937) (Supplemental Fig. S1*A*). As shown, MAGE-D2 was highly expressed in two TNBC cell lines, 231 and 157, but expressed at low levels in 1937 and 468 cells. Thus, a MAGE-D2 knockdown cell model was constructed by transfecting 231 and 157 cells with siRNA against MAGE-D2. In addition, a MAGE-D2 overexpression plasmid was transfected into 468 and 1937 cells. The efficiency of MAGE-D2 knockdown and overexpression was verified by Western blotting (Supplemental Fig. S1, *B* and *C*).

**Fig. 2:**
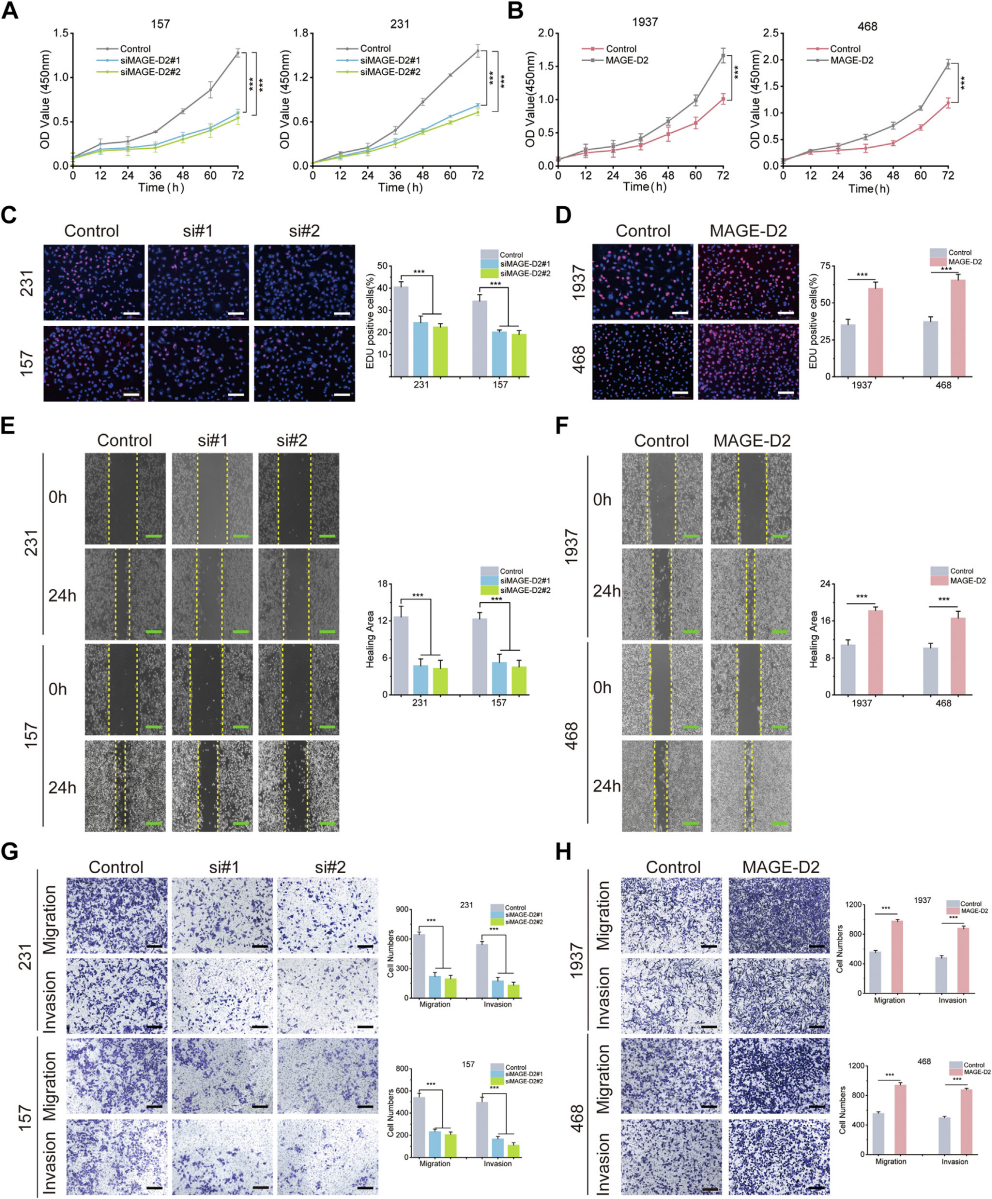
**MAGE-D2 plays a role in cell proliferation and metastasis *in vitro*.***A* and *B*, the growth curves of 231, 157, 1937, and 468 cells using CCK-8 assay. 231 and 157 cells were transfected with siMAGE-D2, 1937 and 468 cells were transfected with MAGE-D2 overexpression plasmid. The control groups were the cells transfected with control siRNA. *C* and *D*, cell proliferation measured by EdU assay. Scale bar represents 100 μm (*white*). *E* and *F*, cell migratory detected by wound healing assay. Scale bar represents 500 μm (*green*). *G* and *H*, cell migratory and invasive abilities assessed by transwell assay. Scale bar represents 200 μm (*black*). Statistical significance was calculated using two-tailed unpaired Student's *t* test in *A* and *B*, *D*, *F*, and *H*. One-way ANOVA test in *C*, *E*, and *G*. Data are presented as mean ± SD, n = 3. ^∗^*p* < 0.05, ^∗∗^*p* < 0.01, and ^∗∗∗^*p* < 0.001. CCK-8, Cell Counting Kit-8; EdU, 5-ethynyl-2-deoxyuridine; MAGE-D2, melanoma antigen family D2.

We then performed Cell Counting Kit-8 and 5-ethynyl-2-deoxyuridine (EdU) assays to detect cell proliferation and carried out transwell and wound healing assays to observe cell migration and invasion abilities in the cell with overexpression and knockdown. The Cell Counting Kit-8 assay demonstrated that MAGE-D2 knockdown suppressed cell proliferation, whereas MAGE-D2 overexpression induced the opposite effect (Fig. 2, *A* and *B*). The results of EdU assays indicated that there were fewer EdU^+^ cells in the MAGE-D2 knockdown group than in the control group (Fig. 2*C*). Correspondingly, MAGE-D2 overexpression promoted cell proliferation, as manifested by an increase in DNA synthesis indicated by increased EdU staining rate (Fig. 2*D*). Furthermore, knockdown of MAGE-D2 also significantly suppressed cell migration and invasion (Fig. 2, *E* and *G*), whereas MAGE-D2 overexpression facilitated cell migration and invasion in transwell chambers (Fig. 2, *F* and *H*). Collectively, these data indicated the promoting role of MAGE-D2 in TNBC proliferation and metastasis *in vitro*.

### MAGE-D2 Regulates TNBC Proliferation and Metastasis *in vivo*

**Fig. 3:**
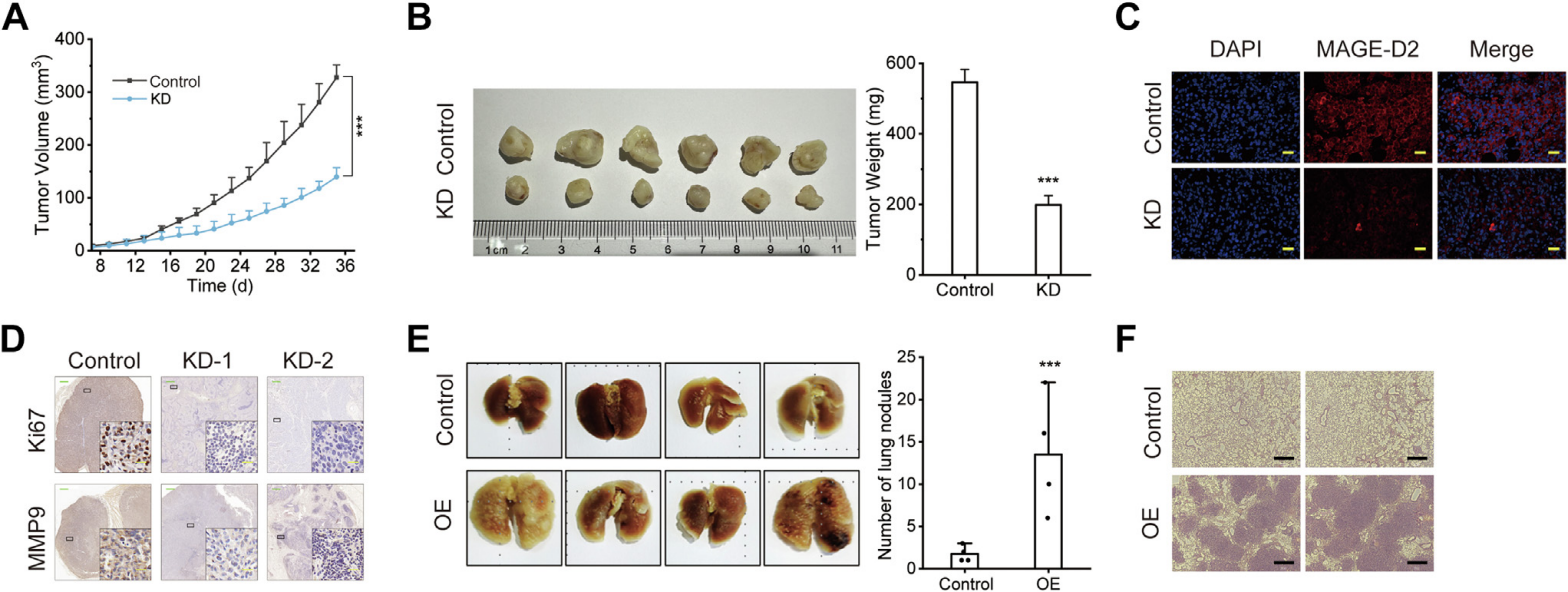
**MAGE-D2 plays a role in cell proliferation and metastasis *in vivo*.***A*, the time course of the average tumor volume of nude mice injected with 231 and MAGE-D2 knockdown 231 cells. The formula for calculating the tumor volume is V = (L × W^2^)/2. *B*, the corresponding images and weights of dissected tumors from the nude mice. *C*, IF staining of MAGE-D2 in tumor tissue. Scale bar represents 20 μm (*yellow*). *D*, representative IHC staining of Ki67 and MMP9 in tumor tissue. Scale bar represents 50 μm (*green*) and 20 μm (*yellow*). *E*, the corresponding images and number of pulmonary surface nodules from the nude mice. *F*, representative images of HE-stained lung sections. Scale bar represents 200 μm (*black*). Statistical significance was calculated using two-tailed Student's *t* test in *A* and *B*. Data are shown as mean ± SD, n = 6 mice per treatment. ^∗^*p* < 0.05, ^∗∗^*p* < 0.01, and ^∗∗∗^*p* < 0.001. L represents the long axis, and W represents the short axis. IF, immunofluorescence; IHC, immunohostochemical; MAGE-D2, melanoma antigen family D2.

To further investigate the underlying role of MAGE-D2 in the proliferation and metastasis of TNBC, nude mouse xenograft models were employed. The results showed that tumors from the mice bearing MAGE-D2 knockdown 231 cells grew more slowly than those from the mice in the control group (Fig. 3, *A* and *B*). MAGE-D2, Ki67, and MMP9 expression levels were reduced in the MAGE-D2 knockdown group (Fig. 3, *C* and *D*). Furthermore, the mice injected with 231 cells stably transfected with the MAGE-D2 overexpression plasmid had significantly more lung metastases than those in the control group (Fig. 3*E*). HE staining showed that the tissue was less organized in the MAGE-D2 overexpression group than in the control group (Fig. 3*F*).

### Label-Free Quantitative Proteomics Revealed Signaling Cascades Downstream of MAGE-D2

**Fig. 4:**
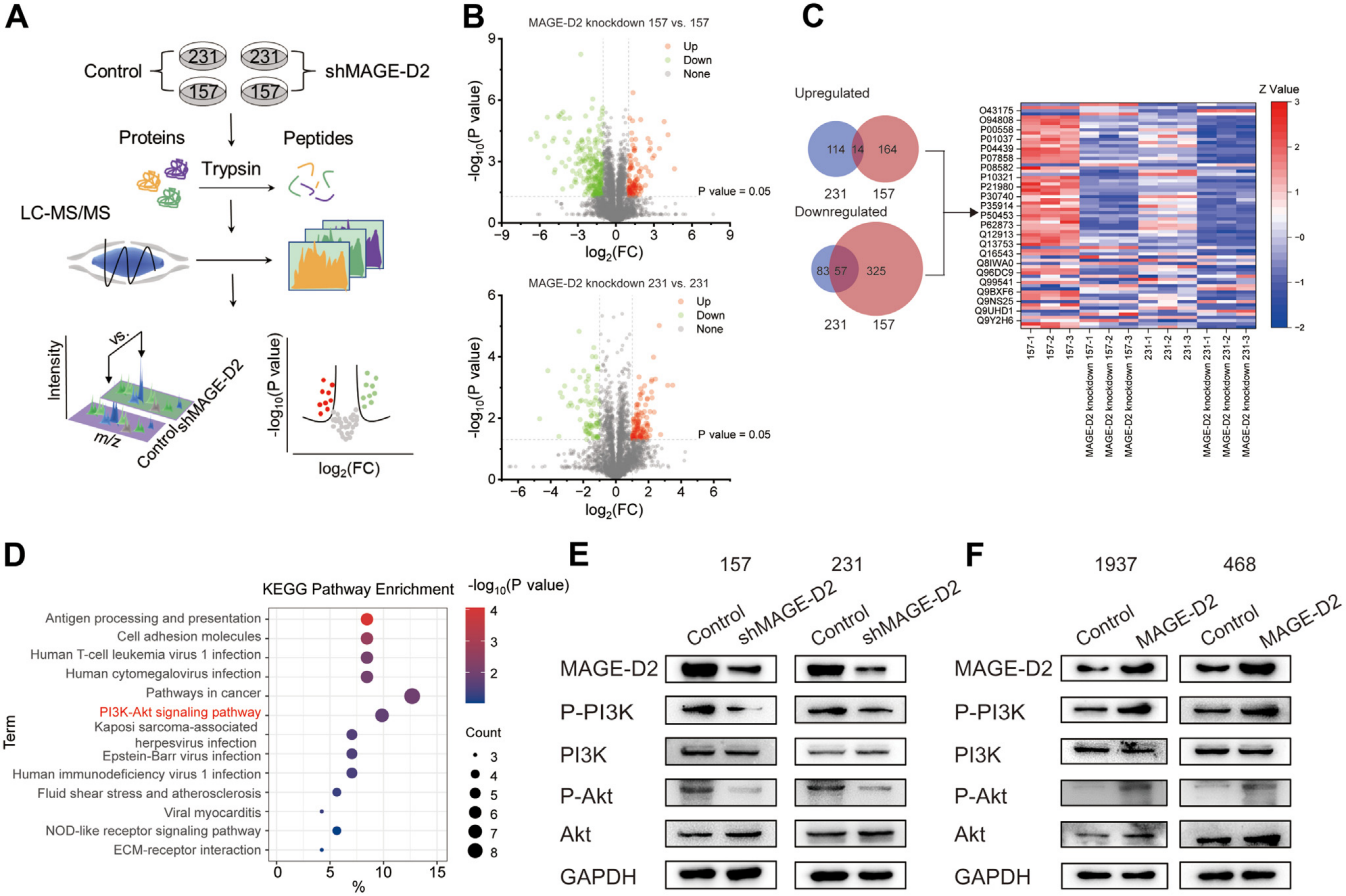
**Proteomic analysis of MAGE-D2 knockdown cells.***A*, workflow of MS-based label-free quantitative proteomic analysis. Two pairs of cells were involved including MAGE-D2 knockdown 231 cells *versus* 231 cells and MAGE-D2 knockdown 157 cells *versus* 157 cells (n = 3). *B*, volcano plot of the identified proteins in two pairs of cells. Student's *t* test was used to identify DEPs (|log_2_FC| >0.6 in relative abundance). *C*, venn diagram of the identified DEPs in two pairs of cells. Heatmap shows the expression of the overlapping DEPs. *D*, KEGG pathways enriched by DEPs in two pairs of cells. *E*, Western blotting of p-PI3K, p-AKT, and total PI3K, AKT in MAGE-D2 knockdown cells and control cells. *F*, Western blotting of p-PI3K, p-AKT, and total PI3K, AKT in MAGE-D2-overexpressing cells and control cells. Proteins identified in three biological replicates for each cell line, with “peptides” ≥3 and “unique peptides” >1, and 1% FDR at peptide and protein levels were considered. DEP, differently expressed protein; FC, fold change; FDR, false discovery rate; KEGG, Kyoto Encyclopedia of Genes and Genomes; MAGE-D2, melanoma antigen family D2; MS, mass spectrometry.

To identify the signaling pathways that mediate the effect of MAGE-D2 in TNBC, we conducted an MS-based label-free quantitative proteomic analysis (Fig. 4*A*). For proteomics studies (12), a stable MAGE-D2 knockdown cell model was constructed by transfecting 231 and 157 cells with shRNA lentiviral particle. Three biological replicates of two pairs of cell groups (MAGE-D2 knockdown 231 *versus* 231, MAGE-D2 knockdown 157 *versus* 157) were used (Supplemental Fig. S1*D*). We detected 5021 proteins in the 157 group (Supplemental Table S2) and 4309 proteins (Supplemental Table S3) in the 231 group. The strong correlation among the three replicates (*R* > 0.95) indicated good repeatability (Supplemental Fig. S2, *A* and *B*). Three biological repeats were well clustered, and the cells were totally segregated, according to the principal component analysis (Supplemental Fig. S2, *C* and *D*). In the volcano plot, 128 proteins in MAGE-D2 knockdown 231 cells were significantly upregulated, whereas 140 proteins were significantly downregulated in MAGE-D2 knockdown 231 cells *versus* control cells. Similarly, 178 upregulated proteins and 382 downregulated proteins were found in MAGE-D2 knockdown 157 cells compared with 157 cells (Fig. 4*B*). There was a significant overlap between these two pairs of cells (14 upregulated DEPs and 57 downregulated DEPs) (Fig. 4*C*, Supplemental Table S4). The DAVID database was used to perform GO term enrichment analysis on these 71 overlapping DEPs. All the DEPs were functionally annotated in the cellular component, biological process, and molecular function categories (Supplemental Fig. S3*A*). More importantly, Kyoto Encyclopedia of Genes and Genomes pathway enrichment analysis demonstrated that MAGE-D2 knockdown induced a decrease in the proteins involved in the PI3K–AKT signaling pathway (LAMC2, ITGAV, HSP90A, LAMB3, CDC37, GNB2, and GNB1) (Fig. 4*D* and Supplemental Fig. S3*B*). Correspondingly, our experiments also demonstrated that PI3K and AKT phosphorylation levels were significantly decreased in MAGE-D2 knockdown cells (Fig. 4*E*) and increased in MAGE-D2-overexpressing cells (Fig. 4*F*).

### IP–MS Revealed MAGE-D2 Interacting Proteins

**Fig. 5:**
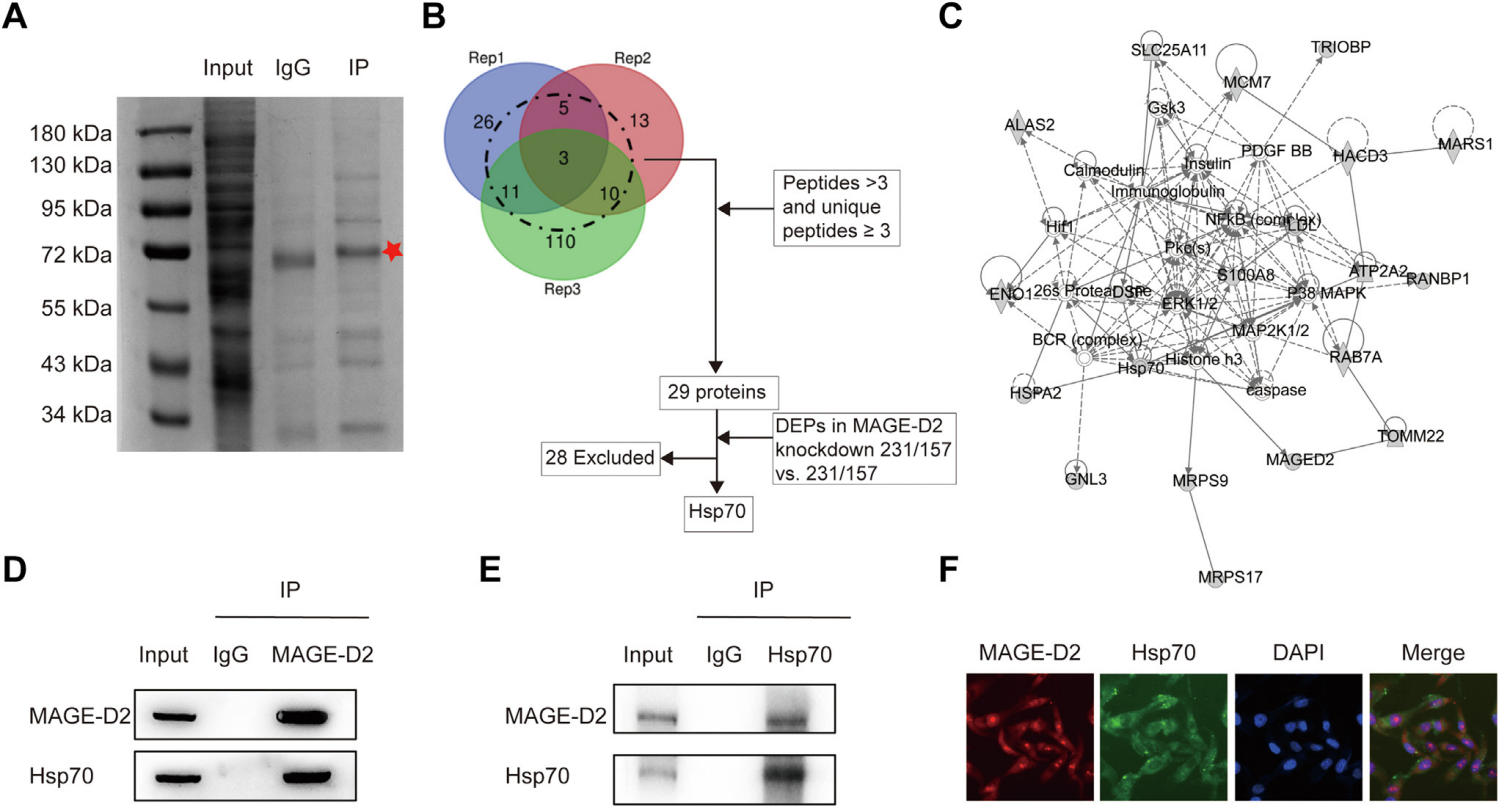
**IP–MS identified MAGE-D2 interacting proteins.***A*, coomassie blue staining of the immunoprecipitates separated by SDS-PAGE after immunoprecipitation using anti-FLAG agarose in MAGE-D2-overexpressing 231 cells. *B*, analysis pipeline to identify the proteins that interacted with MAGE-D2 (n = 3). (1) The proteins pulled down by FLAG were screened. (2) The screened proteins were quantified in at least two biological replicates with “peptides” and “unique peptides” ≥3, and 1% FDR at peptide and protein levels were considered. *C*, IPA result of MAGE-D2 interacting proteins. *D* and *E*, co-IP results show that MAGE-D2 interacts with Hsp70. *F*, double IF staining for MAGE-D2 (*red*) and Hsp70 (*green*) in 231 cells. IgG was used as the control. co-IP, coimmunoprecipitation; FDR, false discovery rate; Hsp70, heat shock protein 70; IF, immunofluorescence; IgG, immunoglobulin G; IPA, ingenuity pathway analysis; IP–MS, immunoprecipitation–mass spectrometry; MAGE-D2, melanoma antigen family D2.

To further investigate the mechanistic roles of MAGE-D2 in the regulation of TNBC, we carried out IP–MS in MAGE-D2-overexpressing 231 cells. The Western blotting and SDS-PAGE results confirmed successful MAGE-D2 purification (Supplemental Fig. S4*A*). A total of 45, 31, and 105 proteins were found in the replicates (Supplemental Table S5). The positive bands were found in Coomassie brilliant blue staining (Fig. 5*A*). Among the 29 MAGE-D2 interacting proteins (Fig. 5*B*, Supplemental Fig. S4*B* and Supplemental Table S6), Hsp70 was the only one with significantly reduced expression in the earlier label-free proteomics results. As shown, the level of interacted Hsp70 significantly decreased with the decrease of MAGE-D2 expression to some extent. The ingenuity pathway analysis results showed that Hsp70 interacts with MAGE-D2, and histone H1.4 (P10412) may be the protein that links Hsp70 and MAGE-D2 (Fig. 5*C*). Western blot analysis demonstrated that Hsp70 expression was consistent with MAGE-D2 expression (Supplemental Fig. S4*C*). The co-IP results also showed that MAGE-D2 was coimmunoprecipitated with Hsp70 and that Hsp70 was coimmunoprecipitated with MAGE-D2 (Fig. 5, *D* and *E*). In addition, MAGE-D2 antibody and Hsp70 antibody can hardly coprecipitate Hsp70 protein and MAGE-D2 protein, respectively, in normal breast epithelial MCF-10A cells, supporting the interaction between MAGE-D2 and HSP70 in TNBC but not in normal cells (Supplemental Fig. S5). Immunofluorescence staining further validated that MAGE-D2 and Hsp70 colocalized in the nucleus and cytoplasm (Fig. 5*F*). These results suggested a potential interaction between MAGE-D2 and Hsp70. Furthermore, we performed XL–MS using a disuccinimidyl suberate crosslinker. But the results did not indicate the existence of the cross-linked peptides of Hsp70 with MAGE-D2 (Supplemental Table S7), implying that the interaction between MAGE-D2 and Hsp70 may not be direct.

### MAGE-D2 Promotes Proliferation and Metastasis by Interacting with Hsp70 Protein and Protecting it From Degradation

**Fig. 6:**
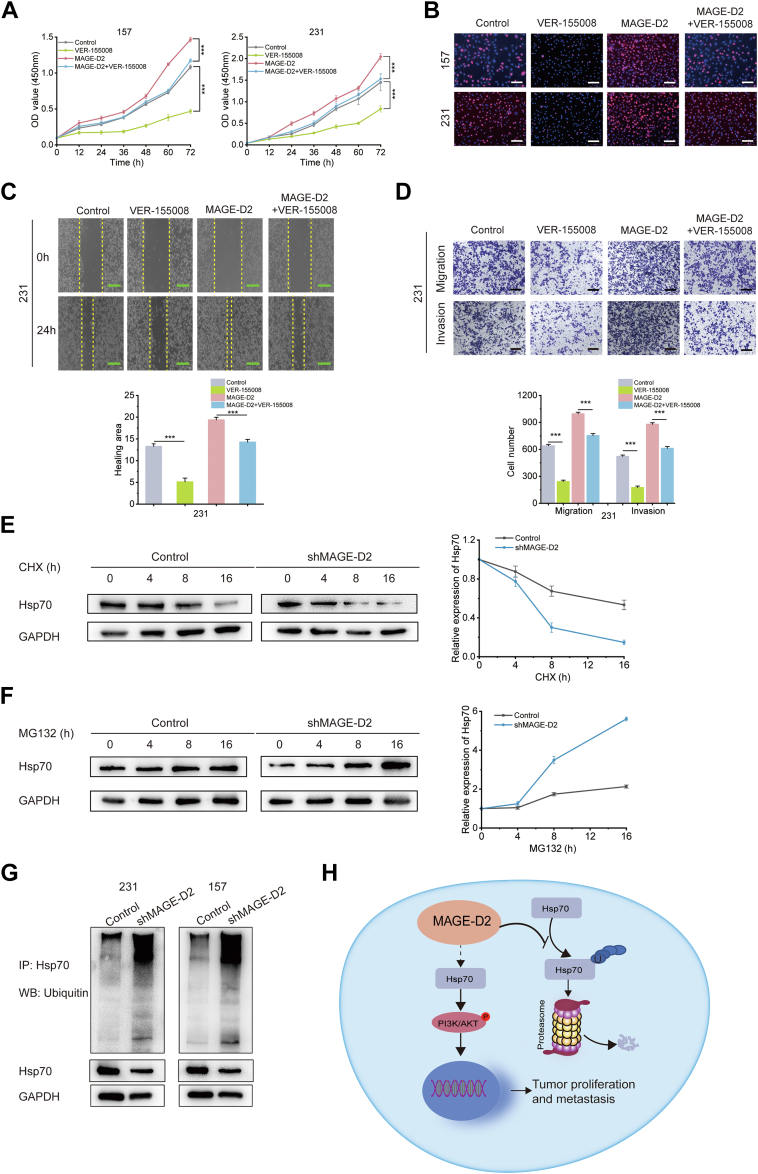
**MAGE-D2 positively regulates Hsp70 expression to promote cell proliferation and metastasis.***A* and *B*, CCK-8 assay and EdU assay of MAGE-D2-overexpressing 231 cells and the cells after the treatment of the Hsp70 inhibitor VER-155008. Scale bar represents 100 μm (*white*) and n = 3. *C* and *D*, the corresponding results of wound healing assay and transwell assay. Scale bar represents 500 μm (*green*) and 200 μm (*black*), n = 3. *E*, Western blotting of Hsp70 protein stability using CHX (cycloheximide) in MAGE-D2 knockdown cells. GAPDH was used as an internal reference. n = 3. *F*, expression of Hsp70 and MAGE-D2 in MAGE-D2 knockdown cells and control cells with the treatment of MG132. GAPDH was used as an internal reference. Relative expression of Hsp70 is the protein expression level of Hsp70 compared with that of GAPDH. *G*, expression of ubiquitinated Hsp70 in MAGE-D2 knockdown cells and control cells with the treatment of MG132. GAPDH was used as an internal reference. *H*, schematic representation of the role of MAGE-D2 in TNBC proliferation and metastasis. MAGE-D2 promotes cell proliferation and metastasis in TNBC by activating Hsp70-PI3K–AKT axis. MAGE-D2 interacts with Hsp70 protein and stabilizes it to prevent its degradation. Statistical significance was calculated using two-tailed Student's *t* test in *A*, *C*, and *D*. Data were represented as mean ± SD. ^∗^*p* < 0.05, ^∗∗^*p* < 0.01, and ^∗∗∗^*p* < 0.001. CCK-8, Cell Counting Kit-8; EdU, 5-ethynyl-2-deoxyuridine; Hsp70, heat shock protein 70; MAGE-D2, melanoma antigen family D2; TNBC, triple-negative breast cancer.

The results showed that the Hsp70 inhibitor VER-155008 dramatically suppressed 231 cell proliferation and viability (Fig. 6, *A* and *B* and Supplemental Fig. S6*A*). In addition, wound healing and transwell experiments indicated that cell proliferation and metastasis caused by MAGE-D2 overexpression could be attenuated by the Hsp70 inhibitor in both 231 cells (Fig. 6, *C* and *D*) and 157 cells (Supplemental Fig. S6, *B* and *C*). RT‒PCR showed that MAGE-D2 had no significant effect on Hsp70 mRNA expression levels (Supplemental Fig. S6*D*). Since the ubiquitin‒proteasome pathway is the main pathway for protein degradation, we used cycloheximide (an inhibitor of protein synthesis) to observe the effect of MAGE-D2 on the stability of Hsp70. The Western blotting results showed that Hsp70 decreased rapidly in MAGE-D2 knockdown cells after cycloheximide treatment, suggesting the impact of MAGE-D2 on Hsp70 expression (Fig. 6*E*). Subsequently, we incubated MAGE-D2 knockdown 231 cells and control cells with MG132 to block protein degradation (Fig. 6*F*). As shown, the reduction in Hsp70 expression in MAGE-D2 knockdown cells was further attenuated by MG132, confirming that MAGE-D2 affects Hsp70 expression through the proteasome pathway. In addition, compared with that in the control group, the Hsp70 ubiquitination level was higher in the MAGE-D2 knockdown group (Fig. 6*G*). Thus, the accumulation of ubiquitinated Hsp70 induced by MG132 was negatively affected by MAGE-D2 expression *via* ubiquitin‒proteasome pathway.

## Discussion

The major findings of the present study are as follows: (1) MAGE-D2 is highly expressed in TNBC; (2) MAGE-D2 overexpression can promote cell proliferation and metastasis by activating the PI3K–AKT pathway; and (3) MAGE-D2 interacts with Hsp70 and inhibits its degradation (Fig. 6*H*). These results suggest that MAGE-D2 may play an important role in TNBC and might thus be a potential therapeutic target. This is the first study to discuss the function and associated mechanism of MAGE-D2 in TNBC.

Proteomic analysis, which was carried out in this study, is an efficient tool to provide more information on individual proteins in the biological context. Most recently, the advancement of proteomics based on MS from straightforward protein sequencing to thorough MS-based biochemical profiling of diseases has yielded a molecular understanding of the processes and pathways underlying cancer biology and an understanding of how these pathways are changed in response to clinical stimuli (13). Among the various proteomic strategies, MS-based proteomics is possible on a large scale since there are public databases, and this method enables a preliminary characterization of diseases. Label-free or isobaric labeling methods are the most commonly used methods and can provide abundant information about the simultaneous identification or quantification of multiple proteins in samples (13). PPI information can also be extracted using IP–MS and XL–MS. In this study, MS-based proteomic strategies revealed the potential role of MAGE-D2 in TNBC.

MAGE-D2, which is a member of the MAGE family, regulates the cell cycle, DNA damage response, proliferation, and apoptosis (14). There are at least 55 closely related proteins in human MAGE. Type I MAGE proteins (MAGE-A∼MAGE-C), which are oncogenic proteins in cancer cells, are frequently generated during embryogenesis and in gamete tissues. The expression of type II MAGE proteins (MAGE-D∼MAGE-H, MAGE-L, and Necdin) varies depending on the tissue (15). Cancer cells overexpress only a few type II MAGE proteins, such as MAGE-D2 (14). Among them, MAGE-A3/6 were found to be required for cancer cell viability (16), and by triggering the epithelial–mesenchymal transition, MAGE-C3 encourages the metastasis of esophageal squamous cancer (17). Moreover, the conserved area known as the MAGE homology domain, which is approximately 200 amino acids long, is present in all MAGE proteins (18). The winged-helix motifs found in this domain enhance the stability of E3 ubiquitin ligases with RING fingers (19). Consistent with bioinformatic analysis of the proteome database, our study also found that MAGE-D2 was highly expressed in TNBC cell and tissue samples. Similar phenomena were observed by Cabezón *et al.* (20), who found a high level of MAGE-A4 (MAGE-D2 family member) expression in TNBC. The laboratory of Zhao *et al.* (21) also found that the expression of another MAGE family member, MAGE-C2, was high in TNBC patients, and individuals with high MAGE-C2 expression had significantly shorter survival periods. Moreover, a few studies have reported high expression of MAGE-D2 in other cancers, such as hepatocellular carcinoma (22), gastric cancer (23), glioma cancer, small intestinal carcinoid (24), and glioma (25). However, the detailed mechanism underlying the association between MAGE and cancer progression and development is still lacking.

Using *in vivo* and *in vitro* experiments, we confirmed the role of MAGE-D2 in TNBC. Our finding is in agreement with previous reports that knockdown of MAGE family proteins could inhibit cell proliferation and that their overexpression can enhance cell migration and invasion (16, 17). Subsequently, label-free quantitative proteomic analysis together with Western blotting showed the association between MAGE-D2 and the PI3K–AKT pathway. Notably, the involvement of the PI3K–AKT pathway in TNBC growth and metastasis has been suggested by previous studies (26, 27). In addition to this pathway, a significant number of DEPs were cell adhesion molecules, including integrin alpha-V, HLA-A, occludin, CD274, intercellular adhesion molecule 1, and HLA-C. Although we did not confirm the role of these adhesion molecules in this study, adhesion molecules have been reported to mediate interactions with other cells and the extracellular matrix, and changes in the expression of cell adhesion molecules directly affect metastasis (28).

The IP–MS and co-IP results indicated that MAGE-D2 interacts with Hsp70. Unfortunately, the results of crosslinking peptide in XL–MS did not show that MAGE-D2 physically crosslinked with Hsp70. However, a significant finding indicated that the peptides crosslinked with MAGE-D2 or Hsp70 belong to the same family, such as MAGE-D3, Hsp40, and Hsp90. There are several possibilities: (1) MAGE-D2 may interact with Hsp70 through other proteins. For example, Laghmani *et al.* (29) previously reported the interaction between MAGE-D2 and Hsp40. Hsp40 and Hsp70 complexes have also been confirmed in a large number of studies. (2) The result may be limited to the arm length of the selected crosslinkers. The arm length of the crosslinkers selected in this study is 11.4 Å; cross-linker length ranges from ∼2.6 Å (*N*,*N*′-carbonyldiimidazole) to ∼42 Å (bulkier protein interaction reporter) (30). Crosslinkers with longer arm lengths can be selected for future research. (3) Since the crosslinkers used in this study were lysine reactive, they might not work as well for those proteins that have fewer lysine residues (31). Further experiments are needed in the future to explore whether MAGE-D2 directly binds to Hsp70.

The Hsp70 protein is a member of the HSP family, and it has been observed in a number of cancer types to be overexpressed and to have aberrant activity (4, 5). In particular, many studies have previously reported the relationship between PI3K–AKT signaling and Hsp70 (32, 33). For example, Liu *et al.* (34) found that blocking the PI3K–AKT signaling pathway *via* Hsp70 inhibition reduced the viability of glioma cells. According to Wan *et al.* (35), the PI3K–AKT–Hsp70–HIF-1 pathway significantly increased the proliferative and angiogenic potential of lung cancer cells exposed to hyperthermia. Therefore, our integrated proteomic analysis indicated the possibility that MAGE-D2 participates in the regulation of the Hsp70-induced activation of the PI3K–AKT signaling network. To date, PI3K inhibitors have assessed in clinical research on TNBC, but single-agent PI3K pathway inhibition has shown limited efficacy in TNBC (36). This study demonstrated that MAGE-D2 might be another potential therapeutic target in the PI3K pathway. Consistent with this speculation, a class of MAGE-A11 inhibitors, 4-aminoquinolines, have exhibited targeted cytotoxicity (37). In the future, a similar strategy could be employed for MAGE-D2 inhibitor discovery.

## Conclusion

This study revealed that MAGE-D2 was an important therapeutic target of TNBC based on an integrated proteomics approach including bioinformatic analysis, label-free quantitative proteomics, and IP–MS technology. Treatment of TNBC may be possible by targeting the MAGE-D2–Hsp70–PI3K–AKT signaling axis. Indeed, there are other MS-based methods, such as electrospray ionization and MALDI (38). In the future, multiple proteomic technologies could be employed for a more comprehensive understanding of the underlying molecular mechanism. Furthermore, functional experiments and clinical studies are needed to confirm the proteomic results.

## Data Availability

The MS proteomics data have been deposited to the iProX Consortium *via* the PRIDE partner repository with the dataset identifier IPX0006040000 (dataset ID: IPX0006040000, login details: shixy sxy195411).

## Supplemental data

This article contains supplemental data (39, 40, 41).

## Ethical Statement

This study was approved by the Institutional Review Board of Jiangsu Cancer Hospital (2019[022]). All the animal experiments were approved by Institutional Animal Care and Use Committee of the Nanjing Medical University (IACUC-2111004) and performed according to the ARRIVE (Animal Research: Reporting of *In Vivo* Experiments) guidelines.

## Conflict of interest

The authors declare no competing interests.
