# Supplementary material for: Proteomic Analysis Revealed the Potential Role of MAGE-D2 in the Therapeutic Targeting of Triple-Negative Breast Cancer
Source: Mol Cell Proteomics. 2023 Dec 20;23(1):100703. doi: 10.1016/j.mcpro.2023.100703 (PMC10835320; doi:10.1016/j.mcpro.2023.100703)
Supplement: Supplemental Data [file mmc1.docx]

**SUPPLEMENTARY MATERIAL**

**Proteomic analysis revealed the potential role of MAGE-D2 in the therapeutic targeting of triple-negative breast cancer**

Xiaoyu Shi^a^, Chunyan Liu^a^, Weimin Zheng^a^, Xiao Cao^a^, Dongxue Zhang^a^, Jianhua Zhu^a^, Xian Zhang^a^, Yun Chen^a,b,c^*

^a^ School of Pharmacy, Nanjing Medical University, Nanjing, 211166, China

^b^State Key Laboratory of Reproductive Medicine and Offspring Health, Nanjing, 210029, China

^c^ Key Laboratory of Cardiovascular & Cerebrovascular Medicine, Nanjing, 210029,

China

***Correspondence:** Dr. Yun Chen (ychen@njmu.edu.cn), School of Pharmacy, Nanjing Medical University, 818 Tian Yuan East Road, Nanjing, 211166, China

**Supplemental Methods**

**Materials**

Primary antibodies against the following target proteins were used: MAGE-D2 (1:1000, HPA031572, Sigma–Aldrich, St. Louis, USA), PI3K (1:1000, 4255, CST, Danvers, USA), Phospho-PI3K (p110α) (1:1000, 9655, CST, Danvers, USA), AKT (1:1000, 9272S, CST, Danvers, USA), Phospho-AKT (Ser 473) (1:1000, 9271S, CST, Danvers, USA), Hsp70 (1:1000, ab182844, Abcam, Cambridge, USA). Secondary anti-rabbit (ab6721, 1:1000) and anti-mouse antibodies (ab6728, 1:1000) for Western blotting were purchased from Abcam (Cambridge, USA). VER-155008 (HY-10941) was ordered from MedChemExpress (Princeton, USA) and the concentration used in the rescue experiments was 1.00 μM. BeyoClick™ EdU Kit, CCK-8 Kit, and BCA reagent was purchased from Beyotime (Shanghai, China). Matrigel and fibronectin were purchased from Sigma-Aldrich (St. Louis, USA). All materials for cell culture and other chemicals were acquired from Thermo Scientific (Logan, USA). Dulbecco’s Modified Eagle Medium (DMEM), Roswell Park Memorial Institute 1640 medium, fetal bovine serum (FBS) and streptavidin-modified beads were obtained from Thermo Scientific (Logan, USA). MEGM kit (Lonza/Clonetics, CC-3150) supplemented with 100 ng/mL cholera toxin was obtained from the Cell Resource Center of the Chinese Academy of Medical Sciences (Shanghai, China). Disuccinimidyl suberate (DSS), dithiothreitol (DTT), indole-3-acetic acid (IAA) were purchased from Thermo Fisher Scientific (Waltham, USA). The sequencing grade trypsin and LysC were ordered from Promega (Madison, WI). Formic acid, trifluoroacetic acid and acetonitrile were ordered from Thermo Scientific (Logan, USA).

**CPTAC dataset**

The proteomic dataset used in this study was PDC000120 (PDC Study ID) and are freely accessible in the CPTAC data portal ^[1]^. Besides tumor samples from 15 TNBC patients, adjacent tissue samples from 18 patients were collected as controls. Raw LC-MS/MS data was processed using the CPTAC Common Data Analysis Pipeline (CDAP) to generate peptide spectrum match reports and protein summary reports (https://pdc.cancer.gov/pdc/study/PDC000120).

**Bioinformatic analysis**

DEPs were chosen using R (Version 4.2.1, Posit), and they were displayed in heatmaps and volcano plots. A protein‒protein interaction (PPI) network was constructed using the STRING database. A Venn diagram was plotted with the “Draw Venn diagram” tool (Draw Venn Diagram (ugent.be)). Thereafter, the DEPs were uploaded to the Kyoto Encyclopedia of Genes and Genomes (KEGG) Database to annotate the pathways enriched in these proteins. Based on the DAVID Bioinformatics Resources (DAVID Functional Annotation Bioinformatics Microarray Analysis (ncifcrf.gov)), the DEPs were subjected to GO annotation based on three categories: biological process, cellular compartment, and molecular function.

**Immunofluorescence (IF) microscopy**

Cells were fixed with 4% paraformaldehyde, permeabilized with 0.3% Triton X-100 in PBS for 15 min at room temperature and blocked with 5% normal goat serum (C0265, Beyotime, Shanghai, China) for 15 min at room temperature. Prior to being incubated with secondary antibodies (1:100), cells were initially treated with anti-MAGE-D2 and anti-Hsp70 antibodies (1:100) at 4 °C overnight. A fluorescence microscope (Nikon, TE200-Μ, Tokyo, Japan) was used to take images.

**Immunohistochemical (IHC) analysis**

Prior to being rehydrated in ethanol, tissue sections were first deparaffinized in xylene. Antigen retrieval was carried out for 15 min in a pH 6.0, 10.0 mM sodium citrate buffer at 100 °C. After then, 3% H_2_O_2_ was used for 10 min to snuff out endogenous peroxidase activity. Sections were blocked with 5% goat serum (C0265, Beyotime, Shanghai, China). The section was incubated with primary anti-MAGE-D2 antibody (1:1000, HPA031572, Sigma–Aldrich, St. Louis, USA) overnight at 4 °C in a humidified chamber, followed by secondary antibodies for 1 h and DAB kit (PR30010, Proteintech, Wuhan, China) for 15 min at room temperature. IHC images were captured using a Pannoramic MIDI slide scanner (3DHISTECH, Budapest, Hungary).

MAGE-D2 expression levels were analyzed using ImageJ software v1.8.0 (National Institutes of Health, Bethesda, USA). Briefly, color images were transformed into 8-bit grayscale images, and a two-threshold technique was then used to calculate the proportion of positively stained region over total tissue area, for each tissue sample. Total tissue area was quantified at a low threshold, while MAGE-D2^+^ areas were quantified at a high threshold. All images were analyzed using fixed threshold values.

**Nude mouse xenograft model**

BALB/c nu/nu mice (female, 5 weeks old) were obtained from the Animal Core Facility of Nanjing Medical University (Nanjing, China, IACUC-2111004). For subcutaneously transplanted tumors in nude mice, 231 and MAGE-D2 knockdown 231 cells were diluted in normal saline containing Matrigel (50%) and then were subcutaneously administered into the BALB/c nude mice's flanks (3×10^6^ cells/100 µL). Tumor volumes were measured every two days until the mice were euthanasia on day 35. Relative tumor volume (%) and relative tumor weight (%) were calculated as described previously ^[2]^. For the lung metastasis model in nude mice, MAGE-D2-overexpressing 231 and control cells were injected through the tail vein into nude mice. The mice were euthanized and the lung tissues were dissected to observe the tumor metastasis after 3 weeks.

**RT-PCR**

RNA was extracted by Trizol (Takara, Japan) according to the instructions. Primer sequences are shown as the following:

(F) MAGE-D2: 5′-GAAAGCCCGAAAGGTGAAGC-3′;

(R) MAGE-D2: 5′-AGCCAACCGAGTCCTTGATG-3′;

(F) Hsp70: 5′-CTTCAACATGAAGAGCGCCG-3′;

(R) Hsp70: 5′-GCAGCAAAGTCCTTGAGTCC-3′;

(F) GAPDH: 5′-AATGGGCAGCCGTTAGGAAA-3′;

(R) GAPDH: 5′- GCCCAATACGACCAAATCAGAG-3′.

**Western blotting**

In brief, the whole-cell lysates were digested, sonicated, and quantified by BCA Protein Quantification Kit in accordance with instructions after the cells were cultivated for 2 days ^[3]^. The proteins were resolved using 10% SDS-PAGE and transferred to a nitrocellulose membrane (0.45 μm, Sartorius, Germany). The membrane was blocked with 5% normal goat serum (C0265, Beyotime, Shanghai, China) at 4 °C overnight followed by incubation with diluted primary antibodies against the following target proteins in sequence at 4 °C overnight: MAGE-D2 (1:1000, HPA031572, Sigma–Aldrich, St. Louis, USA), PI3K (1:1000, 4255, CST, Danvers, USA), Phospho-PI3K (p110α) (1:1000, 9655, CST, Danvers, USA), AKT (1:1000, 9272S, CST, Danvers, USA), Phospho-AKT (Ser 473) (1:1000, 9271S, CST, Danvers, USA), Hsp70 (1:1000, ab182844, Abcam, Cambridge, USA). After each antibody incubation step, the membrane was washed, incubated with secondary antibodies and detected by ECL-chemiluminescence (Pierce^TM^ ECL Kit, Thermo Scientific, Logan, USA).

**Cycloheximide and MG132 experiments**

Cycloheximide (CHX) or MG132 were added to the culture medium at a final concentration of 30.0 μM or 20.0 mM, respectively. Cell lysate was collected at 0, 4, 8, and 16 h after CHX or MG132 treatment. Then, the supernatant was resolved using 10% SDS-PAGE and subjected to Western blotting with anti-MAGE-D2 and anti-Hsp70 antibodies. In addition, cell lysates collected after 6 h treatment with MG132 (10.0 μM) were immunoprecipitated with the anti-Hsp70 antibody, followed by Western blotting using anti-ubiquitin antibody (1:1000, HPA04-263, Sigma–Aldrich, St. Louis, USA).

**CCK-8 and EdU assays for cell growth *in vitro***

In CCK-8 test, 2000 cells per well were planted and cultured for different time. Each well was added with 10 µL of CCK-8 solution and incubated at 37°C for 1 h. Then, the absorbance value at 450 nm was measured by microplate reader (ELx800, BioTek, Vermont, USA).

In EdU test, cells were incubated with EdU solution (C0078S, Beyotime, Shanghai, China) at a final concentration of 10.0 µM for 2 h. Then, the cells were fixed and permeabilized as described above, and incubated with Hoechst 33342 (C1017, Beyotime, Shanghai, China) that was used for nuclear staining, for 10 min, and further with click additive solution at room temperature in the dark for 30 min. Fluorescence microscope was used to take images.

**Transwell and wound healing assays to detect cell migration and invasion *in vitro***

For transwell migration assay, transwell chambers were coated with fibronectin for migration on polycarbonate filters. In a filter with a lower chamber containing 10% FBS and an upper chamber without serum, the cells (5×10^5^) were plated after 12 h of serum-free starvation. After migrating through the filter 12 h later, the cells were dyed with crystal violet and imaged.

For transwell invasion assay, transwell chambers covered with Matrigel on polycarbonate filters were used to measure invasion cells. The cells (1×10^5^) were plated on the upper surface of a filter with the absence or presence of 10% FBS. After culturing at 37 °C for 24 h, the cells that invaded the lower chamber were dyed with 0.1% crystal violet and imaged.

For wound healing test, the cells were planted at a density of 5×10^5^. Use a 100 μL pipette tip to make scratches perpendicular to the plate and add serum-free medium after discarding the culture medium and removing any scratched cells. The cells were imaged after another 24 h of incubation.

**shRNA lentiviral particle, siRNA and** **plasmid transfection**

The shMAGE-D2 lentiviral particle collection (sc-62582-V, Santa Cruz, USA) consists of three distinct shRNA plasmids encoding shRNA as follows:

sc-62581A: GGGAUUCAAUUGAAGGAAATT,

sc-62581B: CGAUGGAAGCGGAUUUGAATT,

sc-62581C: CCUGUGGUUUCUCCUACAATT.

siRNA constructs were purchased from HANBIO Biotechnology (Shanghai, China) with target sequence as follows:

siMAGE-D2#1: ACTTCTGCAGATTCGATATTA,

siMAGE-D2#2: TGAAGTGTGTAACTAACAATT.

A N-terminal 3×Flag-tagged MAGE-D2 overexpression plasmid was purchased from HANBIO Biotechnology (Shanghai, China) with a coding sequence TCTCAAAGGCCCTAATGGCC.

shRNA lentiviral particle, siRNA and plasmid transfections were performed following the manufacturer’s protocols. After the cells were incubated with shRNA lentiviral particle, siRNA or plasmid together with Lipofectamine 3000 (Thermo Fisher Scientific, Logan, USA) at 37 °C for 8 h, the cell medium was changed and the cells were cultivated after 2 days. In the end, MAGE-D2 knockdown 231 cells and MAGE-D2 knockdown 157 cells, MAGE-D2-overexpressing 231 cells, MAGE-D2-overexpressing 468 cells, MAGE-D2-overexpressing 1937 cells were obtained. The transfection efficiency was verified by Western blotting.

**IP-MS analysis**

By incubating 2 µg Flag antibody (1:250, 66008-4-Ig, Proteintech, Wuhan, China) in 500 µL of PBS at 4 °C overnight, MAGE-D2-related proteins in MAGE-D2-overexpressing 231 cells were immunoprecipitated by anti-Flag agarose. The next day, 15 µL of protein A/G agarose beads were added into the immunoprecipitate and the mixture was incubated at 4 °C for 4 h. Then, the beads were boiled at 100 °C for 5 min and the supernatant was collected after centrifuging. Western blotting was used to verify IP. Finally, the sample was analyzed by EASY-nLC™ 1200 HPLC coupled with Orbitrap Fusion Lumos. Raw data were searched by MaxQuant database version 1.6.3.3 (Martinsried, Bavaria, German).

**Co-IP assay and ubiquitination assay**

MAGE-D2-overexpressing 231 cells were lysed in Co-IP cell lysis buffer (P0013J, Beyotime, Shanghai, China) with protease Inhibitor Cocktail (1:1000, P8340, Sigma-Aldrich, St. Louis, USA). Cell lysates were incubated with anti-MAGE-D2 and anti-Hsp70 antibodies at 4 °C overnight followed by protein A/G beads at 4 °C for 4 h. Then, the beads were boiled at 100 °C for 5 min and the supernatant was collected after centrifuging. The sample was then subjected to Western blotting. MAGE-D2 protein was detected with anti-Hsp70 antibody (1:500) and Hsp70 protein was detected with rabbit anti-MAGE-D2 antibody (1:500). Finally, the sample was subjected to Western blotting with anti-ubiquitin antibody (1:1000, ab1586, Sigma-Aldrich, St. Louis, USA).

**Supplementary Figures**


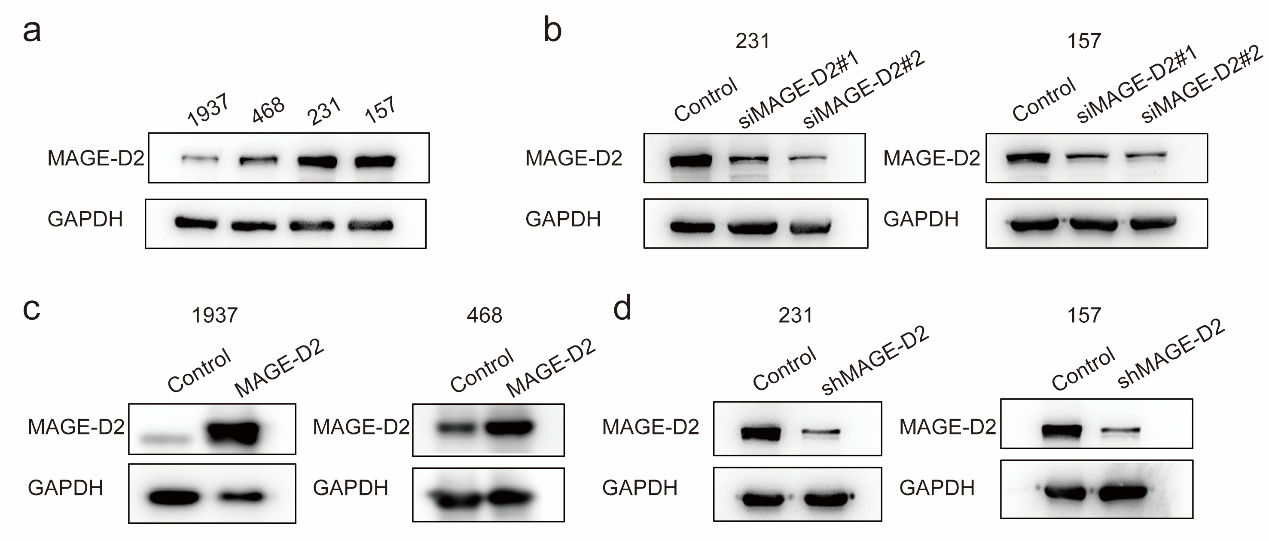


**Figure S1. Evaluation of transfection efficiency by Western blotting.** a. Western blotting of MAGE-D2 expression in four TNBC cell lines (HCC1937, MDA-MB-468, MDA-MB-231, MDA-MB-157); b. Western blotting of MAGE-D2 expression after MAGE-D2 siRNA transfection in 231 cells and 157 cells; c. Western blotting of MAGE-D2 expression after MAGE-D2 overexpression plasmid transfection in 1937 cells and 468 cells; d. Western blotting of MAGE-D2 expression after MAGE-D2 shRNA lentiviral particle transfection in 231 cells and in 157 cells. GAPDH was used as an internal reference.


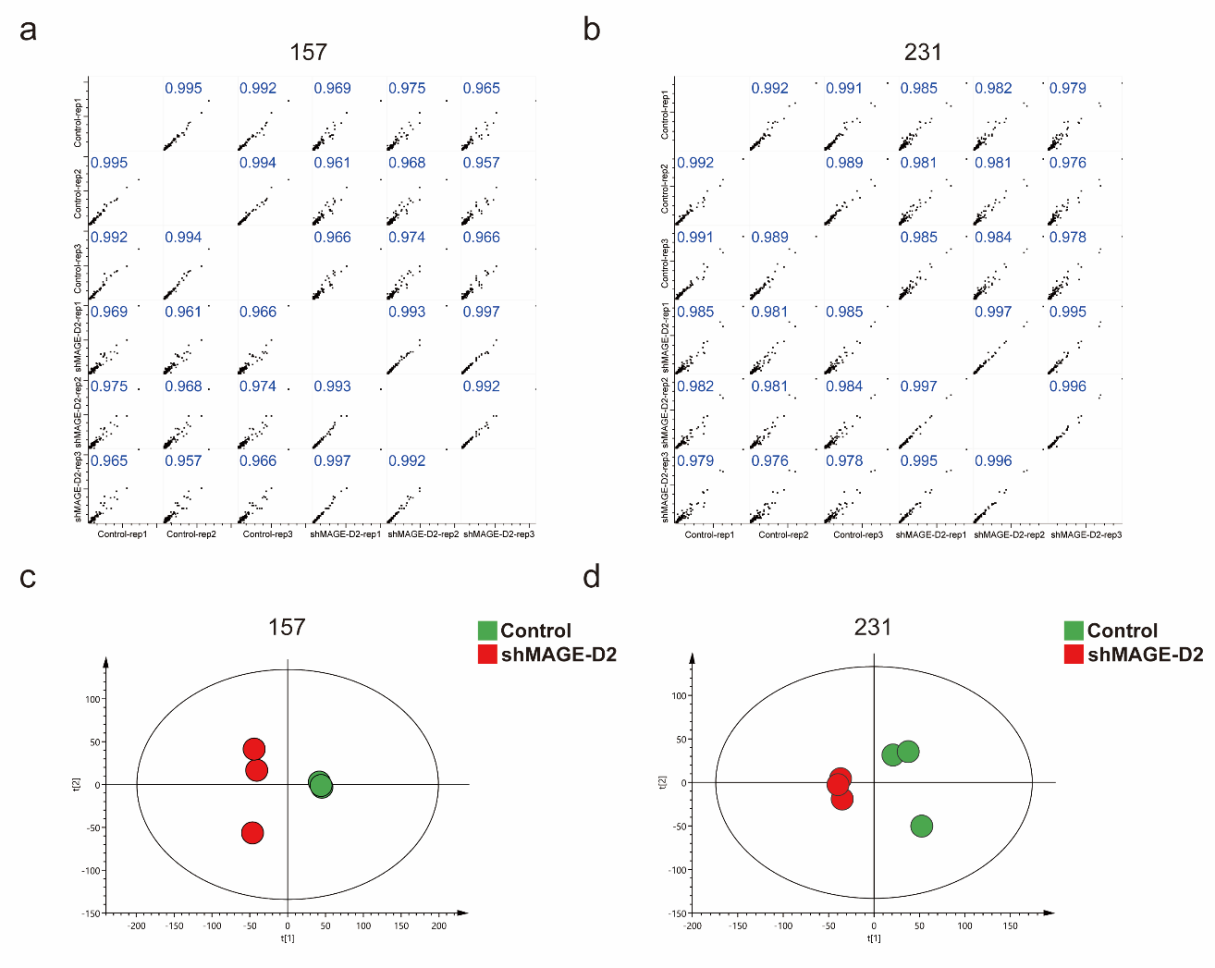


**Figure S2. Reproducibility and clustering of label-free quantitative proteomics data.** a. Reproducibility of the biological replicates in MAGE-D2 knockdown 157 cells and 157 cells; b. Reproducibility of the biological replicates in MAGE-D2 knockdown 231 cells and 231 cells; c. Principal component analysis (PCA) of the biological replicates showing clusters in MAGE-D2 knockdown 157 cells and 157 cells; d. PCA analysis of the biological replicates showing clusters in MAGE-D2 knockdown 231 cells and 231 cells.


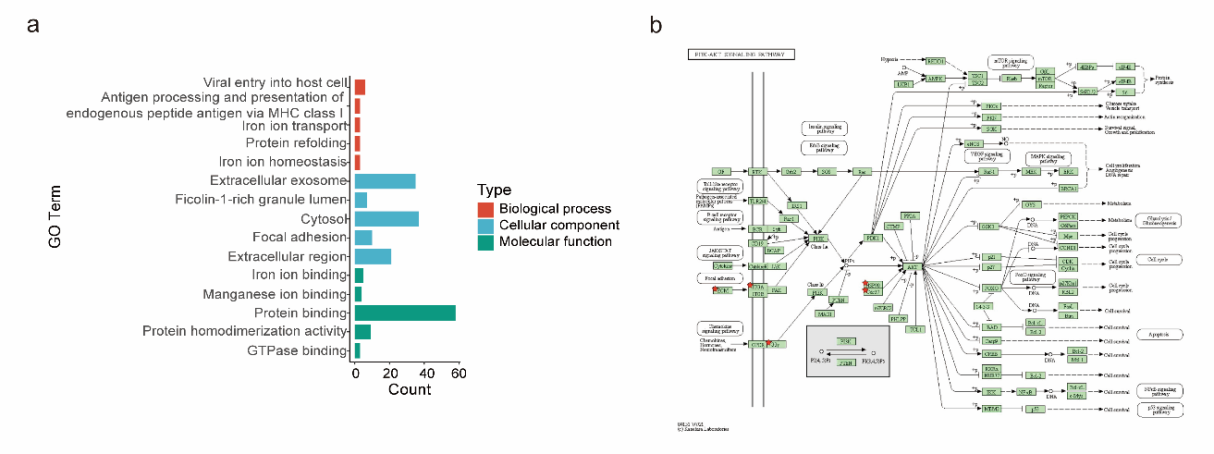


**Figure S3. GO and KEGG enrichment analyses of MS-based label-free quantitative proteomic analysis.** a. GO terms enriched by DEPs in two pairs of cells; b. KEGG analysis showing PI3K/AKT signaling network diagram. Red stars represent DEPs.


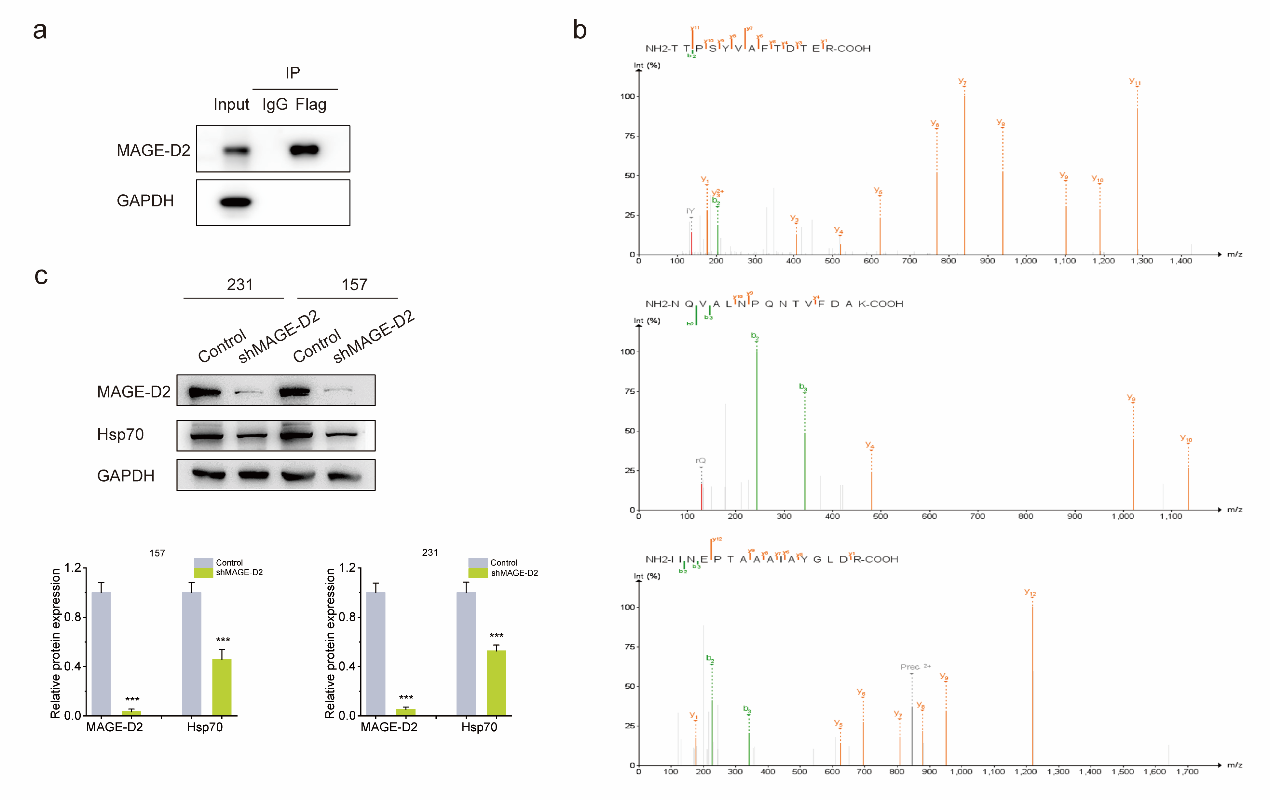


**Figure S4.** a. Evaluation of MAGE-D2 immunoprecipitation using anti-Flag agarose in MAGE-D2-overexpressing 231 cells; b. The detected peptide sequences of Hsp70 after IP-MS; c. Western blotting and relative expression of MAGE-D2 and Hsp70 in MAGE-D2 knockdown 231 and 157 cells. GAPDH was used as an internal reference. Relative expression is the protein expression level of MAGE-D2 or Hsp70 compared to that of GAPDH.


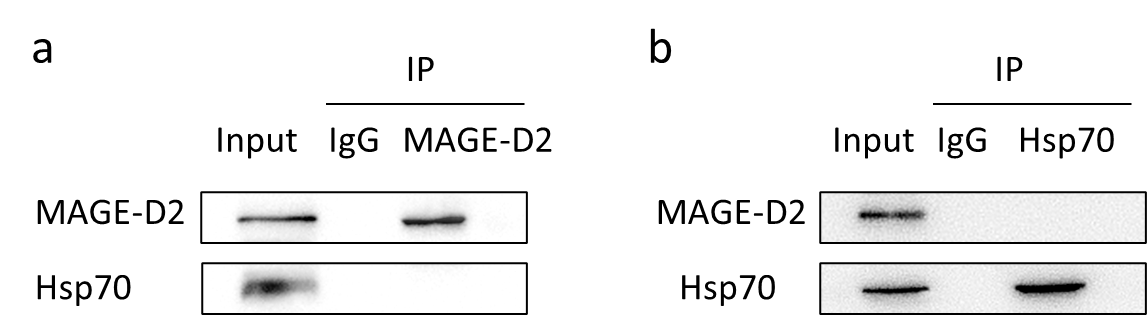


**Figure S5.** **Co‐IP result shows that MAGE-D2 does not interact with Hsp70 significantly in MCF-10A cells.** In detail, a. MAGE-D2 antibody can hardly co-precipitate Hsp70 protein; b. Hsp70 antibody can also hardly co-precipitate MAGE-D2 protein.


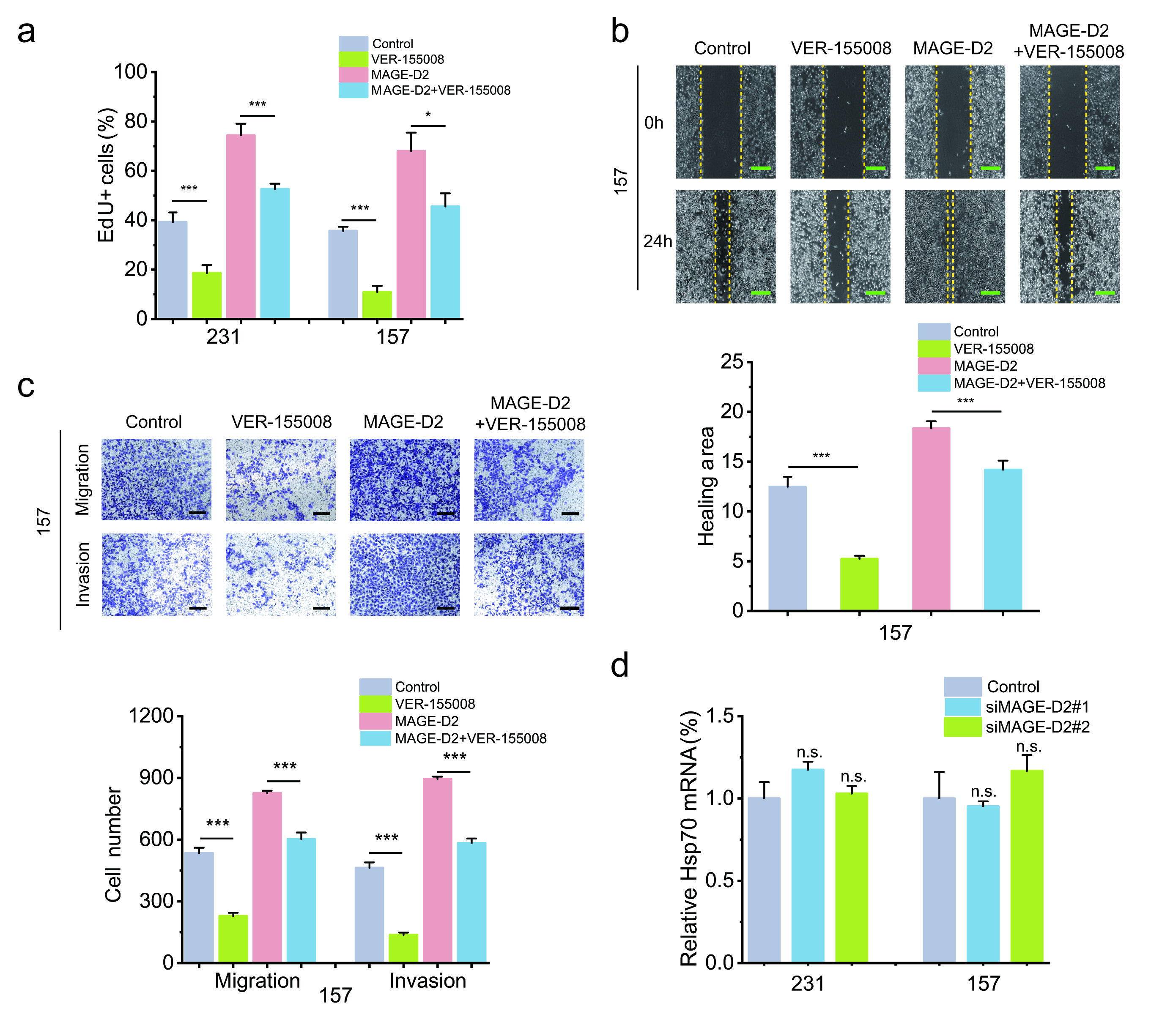


**Figure S6. Cell proliferation and metastasis after the treatment of the Hsp70 inhibitor VER-155008.** a. EdU assay in MAGE-D2-overexpressing cells and control cells with Hsp70 inhibitor treatment; b. Wound healing assay in MAGE-D2-overexpressing cells and control cells with Hsp70 inhibitor treatment. Scale bar: green, 500 μm; c. Transwell assays in MAGE-D2-overexpressing cells and control cells with Hsp70 inhibitor treatment Scale bar: black, 200 μm; d. RT-PCR assays of Hsp70 mRNA expression in MAGE-D2 knockdown 231 cells. n.s., no significance.

**Figure S7. Full-length Western blotting of MAGE-D2 and Hsp70 corresponding to the blots in Figures 6e and 6g.**


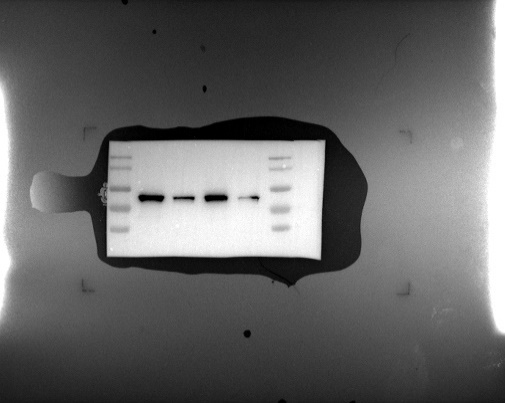


MAGE-D2


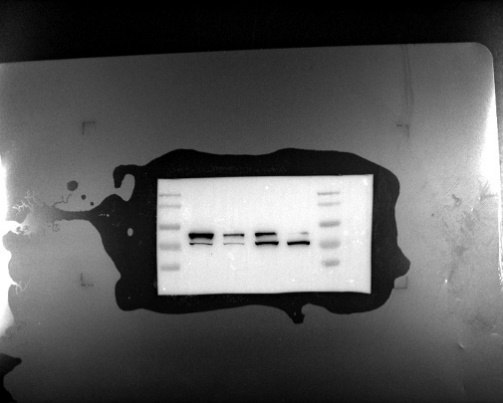


Hsp70

**Supplementary Tables**

**Table S1.** A list of 2,03,835 human protein entries for MaxQuant searching.

**Table S2.** A list of DEPs in MAGE-D2 knockdown 157 cells compared with 157 cells.

**Table S3.** A list of DEPs in MAGE-D2 knockdown 231 cells compared with 231 cells.

**Table S4.** A list of DEPs up- or downregulated in MAGE-D2 knockdown 231 cells vs. 231 cells and MAGE-D2 knockdown 157 cells vs. 157 cells, respectively.

**Table S5.** Lists of MAGE-D2 interacting proteins identified by IP-MS.

**Table S6**. The 29 MAGE-D2 interacting proteins in the label-free proteomics results.

**Table S7.** A list of MAGE-D2 interacting proteins identified by XL-MS and the annotated spectra of the chemically cross-linked peptides in each replicate.

**References**

1. Krug K, Jaehnig EJ, Satpathy S, Blumenberg L, Karpova A, Anurag M, et al. Proteogenomic Landscape of Breast Cancer Tumorigenesis and Targeted Therapy. *Cell*. **2020**;183(5):1436-56.e31.

2. Kodama T, Kochi Y, Nakai W, Mizuno H, Baba T, Habu K, et al. Anti-GPRC5D/CD3 Bispecific T-Cell-Redirecting Antibody for the Treatment of Multiple Myeloma. *Mol Cancer Ther*. **2019**;18(9):1555-64.

3. Kim Y, Ganesan P, Ihee H. High-throughput instant quantification of protein expression and purity based on photoactive yellow protein turn off/on label. *Protein Sci*. **2013**;22(8):1109-17.
